# Qianliexin capsule exerts anti‐inflammatory activity in chronic non‐bacterial prostatitis and benign prostatic hyperplasia via NF‐κB and inflammasome

**DOI:** 10.1111/jcmm.16599

**Published:** 2021-05-13

**Authors:** Linghe Zang, Fangyuan Tian, Yuancheng Yao, Yiran Chen, Yuan Shen, Mingyu Han, Zhaoqing Meng, Shengci Fan, Xinyi Zhang, Tian Cai, Qi Gao, Yuwei Zhang, Jincai Lu

**Affiliations:** ^1^ Institute of Life science and Bio‐pharmaceuticals Traditional Chinese Medicine College Shenyang Pharmaceutical University Liaoning China; ^2^ Institute of Traditional Chinese Medicine Shandong Hongjitang Pharmaceutical Group Co., Ltd Shandong China

**Keywords:** benign prostatic hyperplasia, chronic non‐bacterial prostatitis, inflammasome, inflammation, Qianliexin capsule

## Abstract

Qianliexin capsule (QLX) is a standardized traditional Chinese herbal preparation that has long been used to treat chronic non‐bacterial prostatitis (CNP) and benign prostatic hyperplasia (BPH). This study investigated the anti‐inflammatory activity of QLX in improving lower urinary tract symptoms (LUTS) associated with CNP and BPH. Rat models of CNP and BPH were induced by oestradiol or testosterone (hormonal imbalance) or chemical inflammation (carrageenan). QLX significantly relieved LUTS in CNP and BPH rat model by reducing prostate enlargement, epithelial thickness, pain response time, urine volume and bleeding time, and by improving prostatic blood flow. The expression of the pro‐inflammatory cytokines interleukin (IL)‐1β and tumour necrosis factor (TNF)‐α, the pro‐inflammatory transcription factor nuclear factor kappa‐light‐chain‐enhancer of activated B cells (NF‐κB), and inflammasome components (NLRP3, caspase‐1 and ASC) in CNP and BPH tissues was reduced by QLX addition. QLX treatment was followed by reduced cellular malondialdehyde and increased superoxide dismutase, catalase and glutathione peroxidase activity, consistent with antioxidant activity. Increases in Beclin‐1 expression and the LC3II/I ratio following QLX treatment indicated that autophagy had been induced. QLX relieved LUTS in CNP and BPH rat models by inhibiting inflammation. The underlying mechanisms included inhibition of inflammasome activation, NF‐κB activation, oxidant stress and autophagy.

## 1 INTRODUCTION

The tissue remodelling that accompanies benign prostatic hyperplasia (BPH) consists of nodular overgrowth of the epithelium and growth of fibromuscular tissue within the transition zone and periurethral region.^1^ Chronic non‐bacterial prostatitis (CNP) is a driving force in BPH that is associated with lower urinary tract symptoms (LUTS), including increased urinary frequency, urgency incontinence, nocturia and chronic pelvic pain syndrome that may become serious.^2^ The overall prevalence of CNP is 4.5% to 9%, increases to at least 50% with ageing, and 57.2% of CNP patients progress to BPH with ageing.^3^ The development of prostate cancer is closely associated with chronic prostate inflammation.^4^ The causes of prostate inflammation include infection, primarily by Gram‐negative bacilli, and non‐infectious stimulation, which can be induced by hormonal imbalance.^5^

Inflammasomes can respond to endogenous or exogenous stress signals by initiating an inflammatory cascade in myeloid cells. Assembly of inflammasomes containing nod‐like receptor (NLR) family pyrin domains (NLRP) leads to caspase‐1–dependent release of the pro‐inflammatory cytokines interleukin (IL)‐1β and IL‐18.^6^ In BPH rat models, prostatic inflammation was found to be mediated by NLRP1 inflammasome–induced release of caspase‐1 and downstream release of cytokines IL‐18 and IL‐1β.^7^ Autophagy is a key regulator of tissue homeostasis by inhibiting or promoting the activation of inflammasomes, and inhibition of autophagy in CNP and BPH patients is associated with severe prostatic inflammation.^8^, ^9^ Oxidative stress is another key factor regulating the inflammation response in CNP and BPH.^10^ Oxidative stress results from the formation of reactive oxygen species (ROS) and aberrant enzymatic and non‐enzymatic antioxidative activity. Targeting inflammasomes is one of the reasonable CNP and BPH treatments.

Alpha‐1 adrenergic blockers such as tamsulosin are the most effective, least costly and best tolerated of the available drugs used to relieve LUTS in CNP and BPH patients.^11^ Non‐steroidal anti‐inflammatory drugs (NSAIDs) and 5α‐reductase inhibitors inhibit the synthesis of androgen dihydrotestosterone (DHT) from testosterone and decrease the expression of JM‐27, a serum marker that is highly up‐regulated in symptomatic BPH.^12^ Drug‐related adverse effects can limit the use of those drugs. A standard treatment of CNP and BPH has not yet been established.^13^ Chinese traditional medicine may be able to improve the treatment of CNP and BPH with more acceptable side effects.

Qianliexin capsule (QLX), a standardized traditional Chinese herbal preparation, contains traditional 14 Chinese herbs (Table 1) and has been used in China to treat CNP and BPH for more than 50 years. One clinical study that contains 128 CNP patients reported that QLX improved the quality of life and improved National Institutes of Health‐Chronic Prostatitis Symptom LUTS Index scores, and pro‐inflammation cytokines IFN‐γ and IL‐8 and white blood cell number were significantly reduced in QLX‐treated patients.^14^ Some individual herbals in QLX have anti‐inflammatory and antioxidant activity in various diseases. Myrrha was reported to suppress inflammation in rheumatoid arthritis and neuroinflammation.^15^, ^16^ The anti‐inflammatory activity of extracts of *Salvia miltiorrhiza* is mediated by inhibiting tumour necrosis factor (TNF)‐α and IL‐1β production, and the ethanol extract of *Angelica dahurica* has anti‐inflammatory activity that depends on a cellular redox‐oxidant system.^17^, ^18^ However, the prostatic protective effect and the molecular pharmacology of QLX in CNP and BPH are not obvious. In this study, the anti‐inflammatory activity of QLX, and the role of inflammasomes were investigated in animal models of CNP and BPH.

**TABLE 1:** Composition of QLX capsule

Chinese name	English Name	Plant part	Processing method	1000 capsules (g)	%
Danshen	*Salvia miltiorrhiza* Bunge	Root	Original medicine	84.3	5.17241379
Moyao	*Commiphora myrrha (Nees) Engl*.	Gum	Vinegar making	84.3	5.17241379
Taoren	Prunus persica (L.) Batsch	Seed	Frying	84.3	5.17241379
Chichao	Paeonia lactiflora Pall.	Root	Original medicine	84.3	5.17241379
Honghua	*Carthamus tinctorius L*.	Flower	Original medicine	84.3	5.17241379
Zelan	Lycopus lucidus Turcz. ex Benth.	Aerial part	Original medicine	84.3	5.17241379
Wangbuliuxing	Gypsophila vaccaria (L.) Sm.	Seed	Frying	84.3	5.17241379
Zaojiaoci	Gleditsia sinensis Lam.	Acantha	Original medicine	84.3	5.17241379
Baijiangcao	Patrinia scabiosifolia Link	Whole herb	Original medicine	281	17.2413793
Pugongying	Taraxacum mongolicum Hand.‐Mazz.	Whole herb	Original medicine	281	17.2413793
Chuanlianzi	Melia azedarach L.	Fruit	Original medicine	84.3	5.17241379
Baizhi	Angelica dahurica (Hoffm.) Benth. & Hook.f. ex Franch. & Sav.	Root	Original medicine	84.3	5.17241379
Shiwei	Pyrrosia sheareri (Baker) Ching	Root	Original medicine	140.5	8.62068966
Gouqizi	Lycium barbarum L.	Fruit	Original medicine	84.3	5.17241379

## 2 MATERIALS AND METHODS

### 2.1 Study medications

The major ingredients of QLX including the amounts of individual herbs are listed in Table 1. QLX capsules were manufactured based on the Pharmacopoeia of the People's Republic of China 2020 (p1387‐1388) and the Chinese patent (CN1742903B). QLX capsules (batch numbers: 1 904 009, 1 903 002 and 1 903 004) were provided by the manufacturer, Shandong Hongjitang Pharmaceutical Group Co. Ltd.

### 2.2 Animals and treatment

Sprague Dawley rats were obtained from Liaoning Changsheng Biotechnology (Shenyang, China; Certificate of Conformity: SCXK (Liao) 2015‐0001). Experiments were conducted in accordance with the guidelines of the Animal Care and Use Committee of the Shenyang Pharmaceutical University (Permit No. 211002300042526).

**FIGURE 1:**
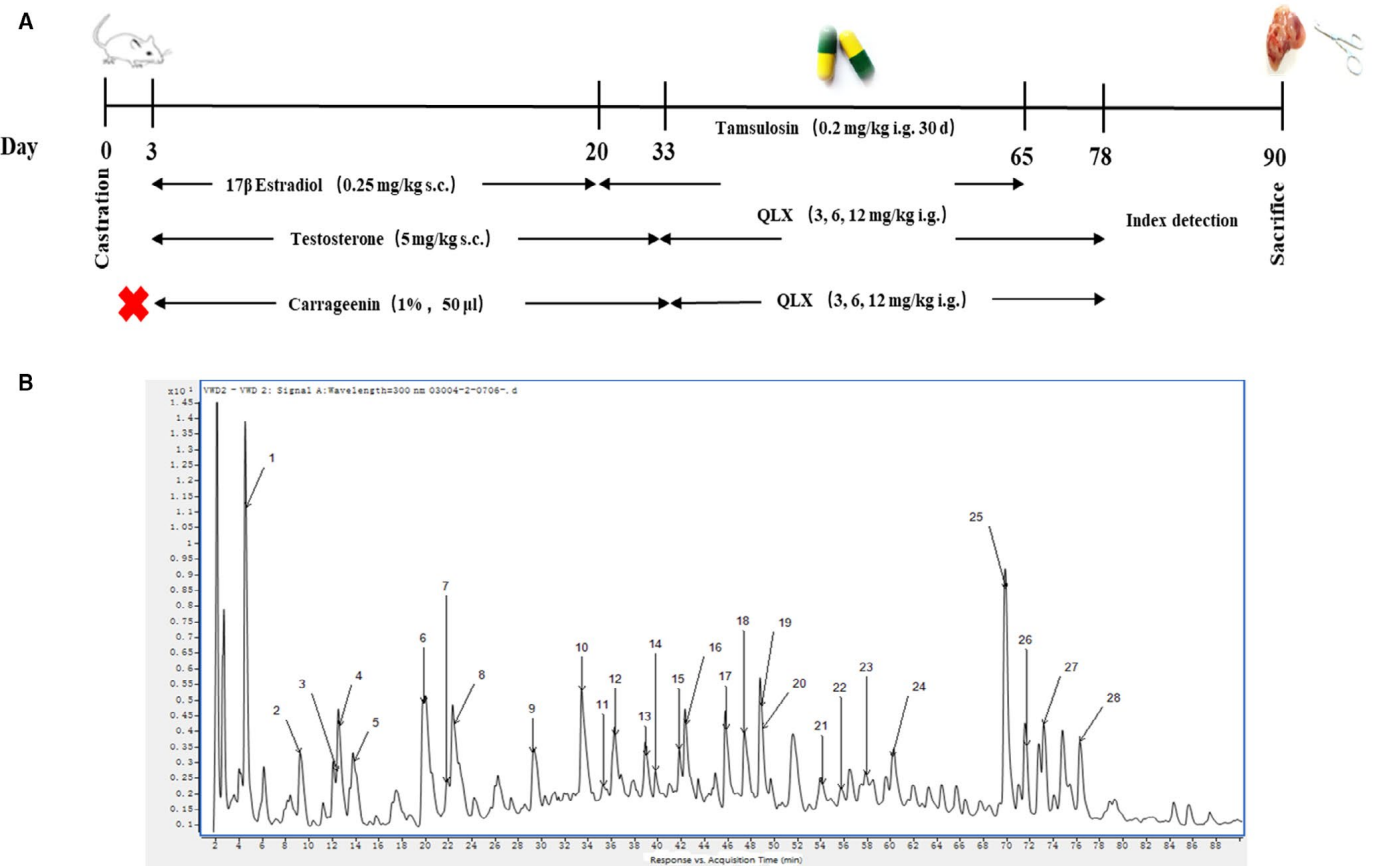
A, Experimental animal protocols that were performed, and UHPLC fingerprint chromatograms of B, the QLX extracts that were obtained. The identified components are shown in Table 1

QLX capsules were dissolved in water at concentrations 10%, 20% and 40% (g/L), and the solutions were administrated to rats by oral gavage three times daily at 3, 6 and 12 g/kg. Tamsulosin (Astellas Pharma) was administered by oral gavage once daily at 0.2 mg/kg (Figure 1A). Male rats (200 ± 20 g) were randomly assigned to a control group treated by sham castration, castration and subcutaneous injection with either 17β‐oestradiol (0.25 mg/kg; Solarbio) for 20 days or testosterone propionate (5 mg/kg; Solarbio) for 33 days beginning on day 3 following surgery or carrageenan (CGN; Solarbio) treatment, in which the prostate was injected intravenously with 1% CGN 50 µL. For drug treatment, control and rat models were given QLX or tamsulosin at the indicated doses for 45 days (Figure 1A).

In the case of SN50 (MedChemExpress) treatment, 2 μg SN50 was intraperitoneally injected into rats every two days from the injection of QLX until the end of experiment.

### 2.3 QLX analysis

Chemical profiling of QLX was performed by UHPLC/Q‐TOF‐MS. A 1.0000 g QLX sample was suspended in 20 mL methanol in a 50‐mL conical flask with a stopper, weighed and extracted in an ultrasonic bath at room temperature for 30 min. After cooling, methanol was added to the starting weight, and the solution was passed through a 0.22‐μm membrane filter before analysis. Chromatography was performed with an Agilent 1290 Series UHPLC System equipped with a binary solvent delivery system, a diode array detector and a column temperature controller. The samples were processed at a column temperature of 25°C with an Agilent Advance Bio peptide mapping column (2.1 × 250 mm, 2.7 μm, PN: 651750‐902). The mobile phase consisted of water with acetonitrile (eluent A) and 0.2% formic acid (eluent B) using a gradient elution mode of 5% A at 0‐5 min, 5%‐10% A at 5‐20 min, 10%‐30% A at 20‐50 min, 30%‐50% A at 50‐65 min, 50%‐80% A at 65‐85 min and 80%‐100% A at 85‐95 min. The flow rate was 0.3 mL·min^‐1^, and the injection volume was 1 μL. Compounds were identified by comparing retention times and spectra, as well as by combining standard solutions and samples, and by fragmentation. Concentration was reported in mg/g of the QLX capsule contents.

### 2.4 Rat model assays

Prostate glands were isolated from BPH and CNP rat models. The prostate index (‰) was calculated as the prostate weight/rat weight ×1,000. Prostate tissue was fixed in 4% formaldehyde for 48 h, embedded in paraffin and sectioned at 4 μm. Sections were deparaffinized, rehydrated with a grafted ethanol series and stained with haematoxylin and eosin. Epithelial thickness and lumen area were quantified by ImageJ. For immunohistochemical staining, serial sections were incubated with primary antibodies against NF‐κB, NLRP3 (Santa Cruz Biotechnology) or anti‐rabbit IgG at 4°C overnight. After washing with phosphate‐buffered saline, sections were incubated with biotin‐labelled secondary antibody (Servicebio).

Pain threshold was recorded as the time in seconds from first contact with a hot plate until a hind paw lick occurred. The 24‐h urine samples were collected from individual rats placed in metabolic cages after drug delivery cycle. Blood clotting was reported as the time in seconds needed for coagulation of 50 µL blood placed on a glass slide. Prostate blood flow was measured with a laser speckle contrast imager (MoorFLPI‐2, Moor Instruments) in anaesthetized rats following surgical exposure.

### 2.5 Western blot and ELISA

For Western blot assays, tissue samples were homogenized and then lysed in NETN buffer. Anti‐NF‐κB, IL‐1β, TNF‐α, NLRP3, ASC, caspase‐1, Beclin‐1, LC3, Atg4B, p62 and LAMP2 (SCBT) were the primary antibodies. Protein expression was visualized by enhanced chemiluminescence (ECL) of horseradish peroxidase–conjugated (HRP) secondary antibodies. IL‐1β, TNF‐α, MDA, SOD, GSH and CAT concentrations or activities were determined with ELSA kits following the manufacturer's (Youxuan Bio or Nanjing Jiancheng) instructions.

### 2.6 Immunofluorescence

The specimen of frozen tissue was fixed in 4% formaldehyde, blocked and permeabilized in 5% serum‐PBS buffer with 0.3% Triton × ‐100. Primary antibody against LAMP2 and Ly6G (SCBT) was incubated overnight at 4°C. FITC‐conjugated secondary antibody was used, and DAPI was used for counterstaining.

### 2.7 Statistical analysis

Data were reported as means ±standard deviation (SD). The significance of between‐group differences of means was determined by Student's t test. *P*‐values <0.05 were considered significant.

## 3 RESULTS

### 3.1 Major components of QLX

UHPLC‐Q‐TOF‐MS identified and determined the concentrations of 28 major chemical components in QLX solutions (Figure 1B and Table 1). Two types of compounds predominated, 1‐17, 20 and 22 were polyphenols, most of which included 1‐2 caffeoyl segments in the structures. Compounds 18, 19, 21 and 23‐27 were coumarins derived from *Angelica dahurica* (Table 2). Chemical structures of the 28 components are listed in the supplement figure.

**TABLE 2:** Major chemical components of QLX

Peak No.	Compound	Retention time (min)	Molecular formula	Theoretical value [M‐H]^‐^ (m/z)	Measured value [M‐H]^‐^ (m/z)	Theoretical value [M + H]^+^ (m/z)	Measured value [M + H]^+^ (m/z)	Content (mg/g)	RSD (%)
1	Gallic acid	4.985	C_7_H_6_O_5_	169.0142	169.0143			11.8102	2.40
2	Protocatechuic acid	9.745	C_7_H_6_O_4_	153.0193	153.0192			7.4144	1.21
3	Caftaric acid	12.823	C_13_H_12_O_9_	311.0409	311.0408			0.9175	1.34
4	Neochlorogenic acid	13.109	C_16_H_18_O_9_	353.0878	353.0867			11.5105	2.27
5	Protocatechualdehyde	13.848	C_7_H_6_O_3_			139.0390	139.0386	0.3877	1.11
6	Chlorogenic acid	20.627	C_16_H_18_O_9_	353.0878	353.0881			45.1841	1.77
7	Caffeic acid	22.746	C_9_H_8_O_4_	179.0350	179.0346			4.3270	0.77
8	Cryptochlorogenic acid	23.200	C_16_H_18_O_9_	353.0878	353.0872			29.4802	0.99
9	4‐Hydroxycinnamic acid	30.180	C_9_H_8_O_3_	163.0401	163.0402			2.3235	1.31
10	Chicoric acid	34.267	C_22_H_18_O_12_	473.0725	473.0716			2.1110	2.98
11	Taxifolin	36.050	C_15_H_12_O_7_	303.0510	303.0504			1.8699	2.01
12	Isoquercitrin	37.143	C_21_H_20_O_12_	463.0882	463.0872			33.4400	1.55
13	Isochlorogenic acid B	39.397	C_25_H_24_O_12_	515.1195	515.1183			0.8676	1.21
14	Isochlorogenic acid A	40.338	C_25_H_24_O_12_	515.1195	515.1183			2.2490	1.54
15	Isochlorogenic acid C	42.289	C_25_H_24_O_12_	515.1195	515.1195			1.3349	1.26
16	Rosmarinic acid	43.130	C_18_H_16_O_8_	359.0772	359.0770			12.4849	0.91
17	Salvianolic acid B	46.208	C_36_H_30_O_16_	717.1461	717.1453			11.5584	1.93
18	Oxypeucedanin hydrate	47.754	C_16_H_16_O_6_			305.102	305.1021	1.5817	1.11
19	Byakangelicin	49.066	C_17_H_18_O_7_			335.1125	335.113	1.7135	1.19
20	Salvianolic acid A	49.421	C_26_H_22_O_10_	493.1140	493.1140			20.8320	1.12
21	Xanthotoxin	54.078	C_12_H_8_O_4_			217.0495	217.0492	0.3422	1.35
22	Kaempferol	55.861	C_15_H_10_O_6_			287.055	287.0553	0.0709	0.98
23	Bergapten	58.031	C_12_H_8_O_4_			217.0495	217.0496	0.5281	1.49
24	Oxypeucedanin	63.345	C_16_H_14_O_5_			287.0914	287.0917	1.4303	1.68
25	Imperatorin	71.418	C_16_H_14_O_4_			271.0965	271.0963	7.0624	0.59
26	Phellopterin	89.986	C_17_H_16_O_5_			301.1071	301.1071	3.9297	1.37
27	Isoimperatorin	92.476	C_16_H_14_O_4_			271.0965	271.097	1.8017	1.31
28	Cryptotanshinone	94.376	C_19_H_20_O_3_			297.1485	297.1492	0.1191	1.39

### 3.2 Effects of QLX administration on CNP and BPH induced by hormonal imbalance

**FIGURE 2:**
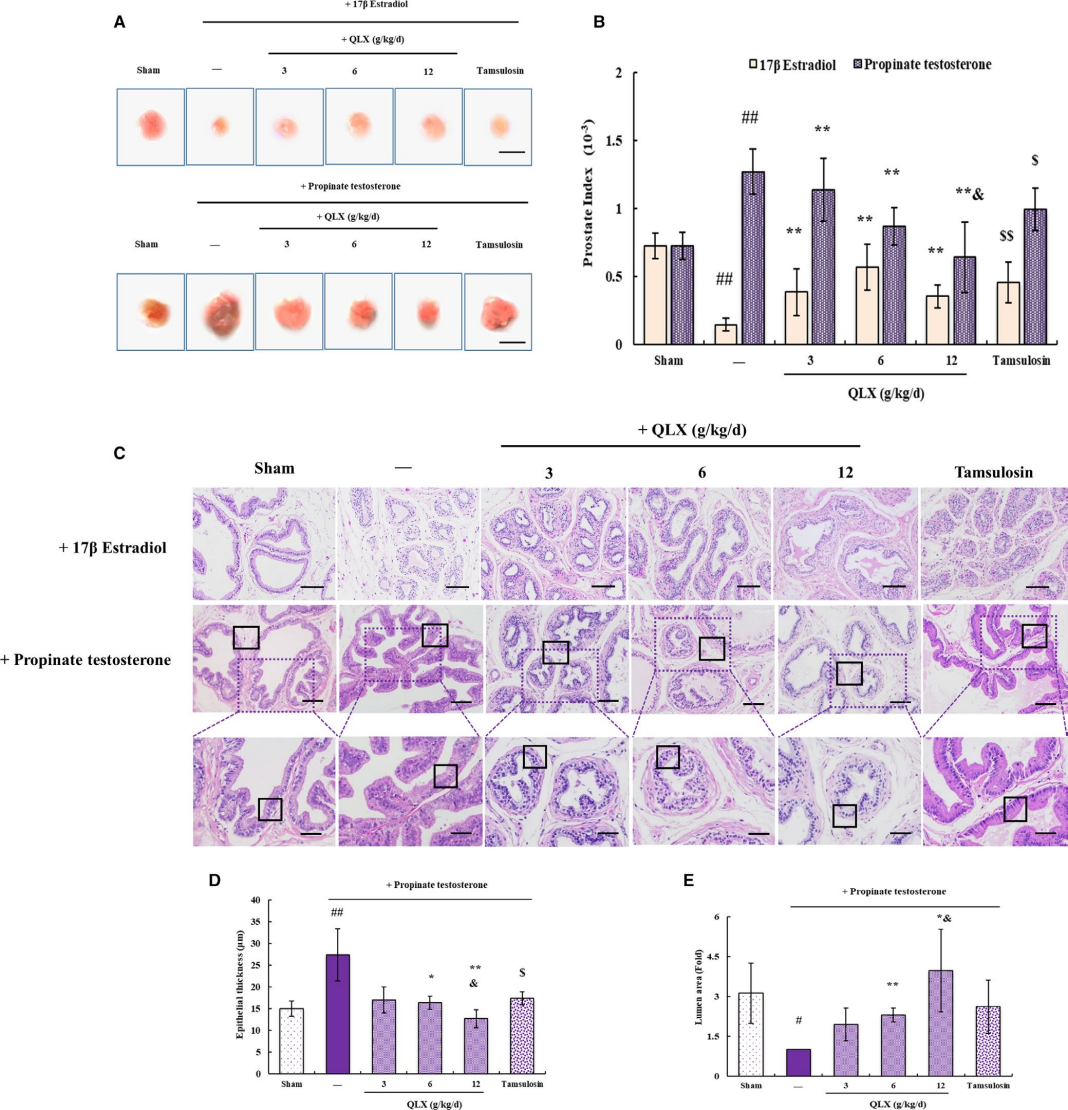
QLX exerts protective effect in hormonal imbalance–induced CNP and BPH. A, Representative photographs showing the size of prostate glands. B, The prostate index was based on the weight of prostate relative to the rat bodyweight. C, Representative HE staining of prostate tissue showing D, the thickness of the epithelium and E, the luminal area. Castrated rats were treated with oestradiol or testosterone to induce CNP and BPH. Black frame in the middle panel indicates the luminal area. Black frame in the lower panel indicates the thickness of the epithelium. The typical enlarged photographs are shown in the lower panel as indicated. QLX was administered at the indicated dose for 45 days. Tamsulosin was used as a positive control. Control rats were treated by sham surgery. Data are means ± SD, n = 6. ^#^ *P* < .05, ^##^ *P* < .01 (sham vs. oestradiol or testosterone‐treated rats); ^*^ *P* < .05, ^**^ *P* <.01 (QLX‐treated vs. oestradiol or testosterone‐treated rats); ^$$^ *P* < .01 (tamsulosin‐treated vs oestradiol or testosterone‐treated rats); ^&^ *P* < .05 (tamsulosin‐treated vs. QLX‐treated rats)

**FIGURE 3:**
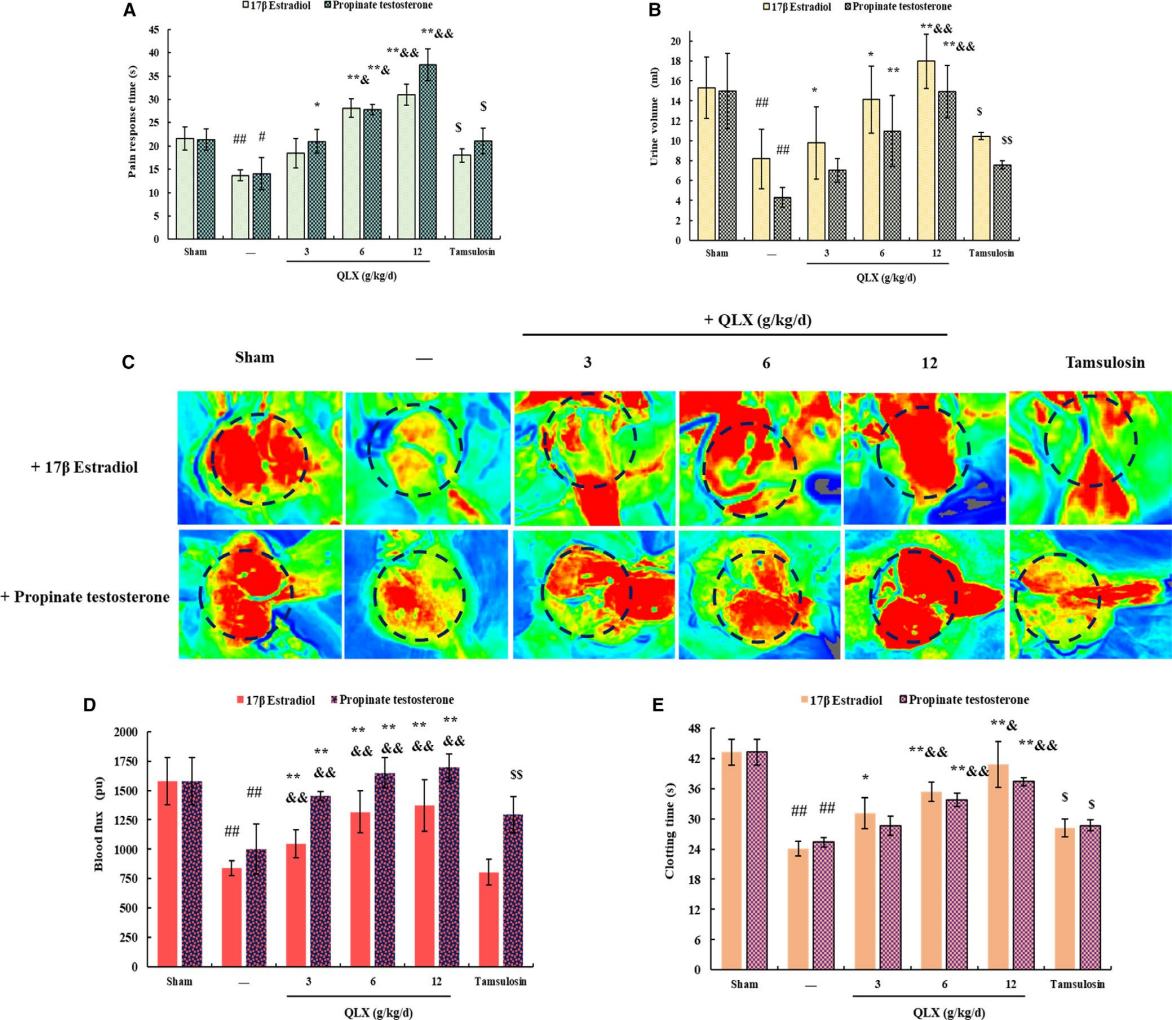
QLX reversed hormonal imbalance–induced LUTS. A, Pain response was measured with a hot plate. B, Urine was collected from rats kept in metabolic cages for 24 h. C and D, Prostate blood flow was measured with a laser speckle contrast imager. Representative photographs of rats in each group are shown. E, Clotting times are shown. The rats were treated as described in Figure 2. Data are means ± SD, n = 3. ^#^ *P* < .05, ^##^ *P* < .01 (sham vs. oestradiol‐ or testosterone‐treated rats); ^*^ *P* < .05, ^**^ *P* < .01 (QLX‐treated vs. oestradiol‐ or testosterone‐treated); ^$^ *P* < .05, ^$$^ *P* < .01 (tamsulosin‐treated vs. oestradiol‐ or testosterone‐treated rats), ^&^ *P* < .05 (tamsulosin‐treated vs. QLX‐treated rats)

Hormonal imbalance is a key stimulator of CNP and BPH, which were induced in this study by injection of oestradiol or testosterone. QLX was administered to rat models at 3, 6 and 12 g/kg/d for 45 days. Tamsulosin, α1‐adrenergic blocking agent used clinically to treat BPH, was used as a positive control (Figure 1A). The prostate index indicated prostate enlargement and was measured after QLX treatment of oestradiol‐ or testosterone‐treated rats. Testosterone significantly increased prostate index. Tamsulosin was significantly reversed testosterone–induced prostate enlargement. QLX reduced the prostate index in a dose‐dependent manner, and the effect of QLX was better than tamsulosin at high doses (Figure 2A and B). This indicated that QLX reduced prostate enlargement in CNP and BPH. Oestradiol significantly repressed the prostate growth, and QLX and tamsulosin also derepress the size of prostate (Figure 2A and B). HE staining indicated thickness of the epithelium and involution of epithelial cells into the lumen were increased in both oestradiol‐ and testosterone‐treated rats. QLX reverses the abnormal prostate gland histology in the CNP and BPH rat models (Figure 2C), with a significant reduction in the increase in the epithelial thickness and decrease in luminal area in the prostate glands of testosterone‐treated rats in response to 6 and 12 g/kg/d QLX (Figure 2D and E). The results indicated that QLX may have dual effects in regulating CNP and BPH induced by hormonal imbalance. Both oestradiol and testosterone significantly shortened pain response time compared with controls; QLX significantly increased pain response time of rat models in a dose‐dependent manner (Figure 3A). The decrease in urine volume that accompanied CNP and BPH was significantly inhibited by QLX (Figure 3B), indicating that QLX relieved urinary frequency and chronic pelvic pain syndrome.

Reduced prostatic blood flow has been reported in CNP and BPH patients, and increased prostatic blood flow has been reported to relieve LUTS in CNP and BPH patients.^19^ Prostatic blood flow was significantly increased by QLX in rats with CNP and BPH induced by either testosterone or oestradiol (Figure 3C and D). Inflammation initiates clotting, decreases the activity of natural anticoagulant mechanisms and impairs the fibrinolytic system.^20^ The significant increase in clotting time in QLX‐treated rats may indicate that QLX reduced prostatic inflammation (Figure 3E). Tamsulosin also slightly and significantly reversed LUTS in hormonal imbalance–induced CNP and BPH, and high amount of QLX exerted better effect than tamsulosin. The results indicated that QLX significantly reversed LUTS in CNP and BPH induced by hormonal imbalance and had a dual effect on regulating prostatic hyperplasia.

### 3.3 QLX has anti‐inflammatory activity in CNP and BPH induced by hormonal imbalance

**FIGURE 4:**
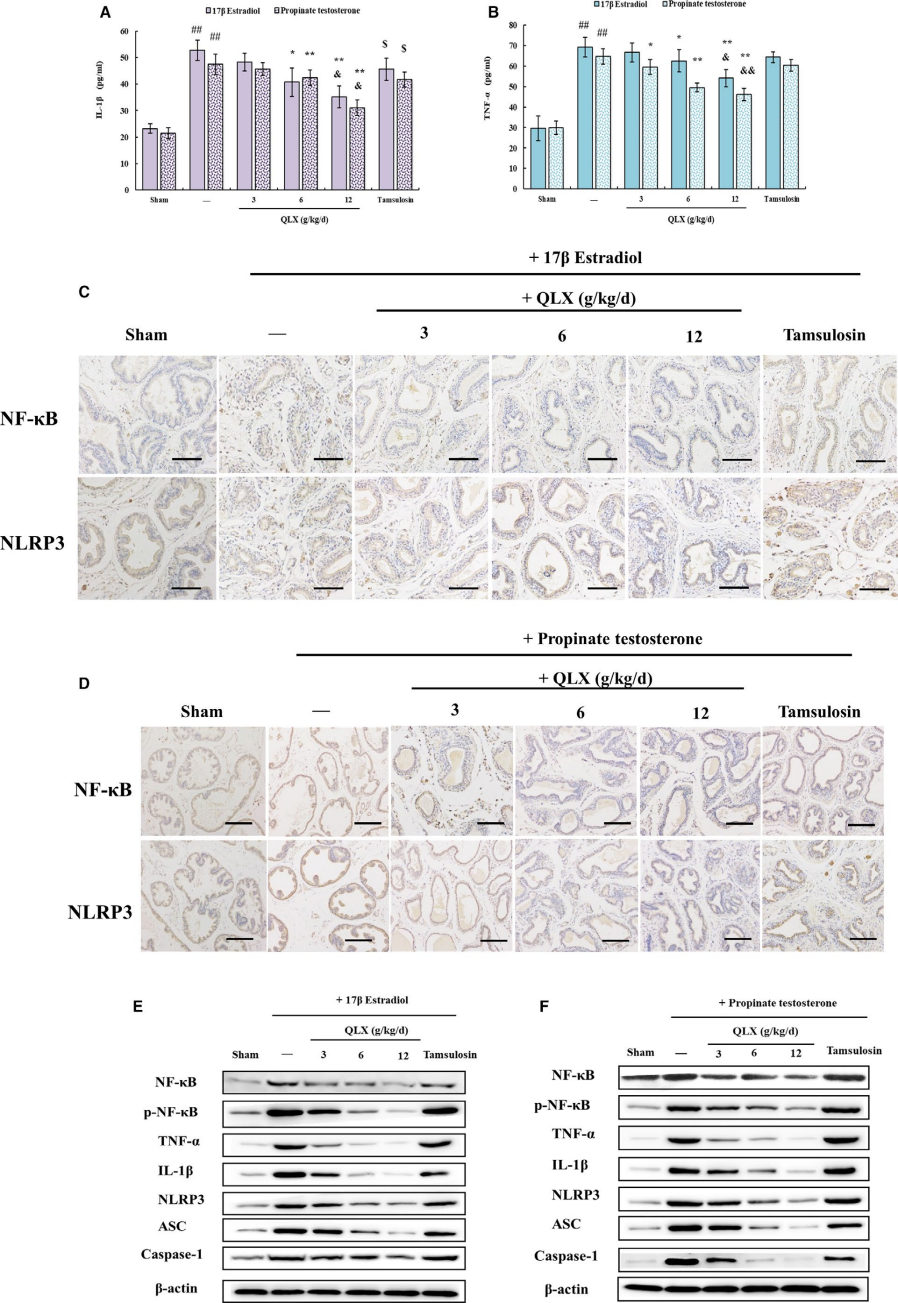
QLX exerts anti‐inflammatory activity in hormonal imbalance–induced CNP and BPH. IL‐1β A, and TNF‐β B, were quantified by ELISA in prostate tissue. NF‐κB C, and NLRP3 D, expressions were assayed by IHC. The expression of other proteins in prostate tissue was assayed in Western blots. The rats were treated as described in Figure 2. Data are means ± SD, n = 3. ^#^ *P* < .05, ^##^ *P* < .01 (sham vs. oestradiol‐ or testosterone‐treated rats); *P < .05, **P <.01 (QLX‐treated vs. oestradiol‐ or testosterone‐treated); ^$^ *P* < .05 (tamsulosin‐treated vs. oestradiol‐ or testosterone‐treated rats); ^&^ *P* < .05 (tamsulosin‐treated vs. QLX‐treated rats)

The infiltration of inflammatory cells seen in HE‐stained prostate tissue in both oestradiol‐ and testosterone‐treated rats was significantly reduced by QLX (Figure 2C). IHC and Western blotting revealed increased expression of the pro‐inflammatory cytokines IL‐1β and TNF‐ α in prostate tissue from oestradiol‐ and testosterone‐treated rats, and the increases were inhibited by QLX (Figure 4A, B, E and F). NF‐κB is a transcription factor that promotes the expression of IL‐1β and TNF‐α.^21^ NF‐ κB expression was increased in prostate tissue from rat models, and the increase was inhibited in a dose‐dependent manner by QLX (Figure 4C‐F). Phosphorylation results in nuclear translocation and accumulation of NF‐κB,^22^ and was decreased by QLX in prostate tissue from rat models (Figure 4E and F). The results indicated that the effects of QLX in rat models were mediated by its anti‐inflammatory activity.

The release of IL‐1β by NLRP3 inflammasomes is mediated by caspase‐1 activity.^6^ IHC confirmed that NLRP3 expression was increased in both oestradiol‐ and testosterone‐treated prostate tissue, and the increase was inhibited by QLX (Figure 4C, D, E, and F). Expression of both caspase‐1 and ASC (the adaptor molecule apoptosis‐associated speck‐like protein containing a CARD), a complex multiprotein inflammasome component, was significantly reduced by QLX in a dose‐dependent manner (Figure 4E and F). The results showed that inflammasome activation and NF‐κB activation of pro‐inflammatory cytokine expression and release were blocked by QLX in prostate tissue from hormone imbalance–induced rat models.

### 3.4 QLX has anti‐inflammatory activity in CGN‐induced CNP and BPH

**FIGURE 5:**
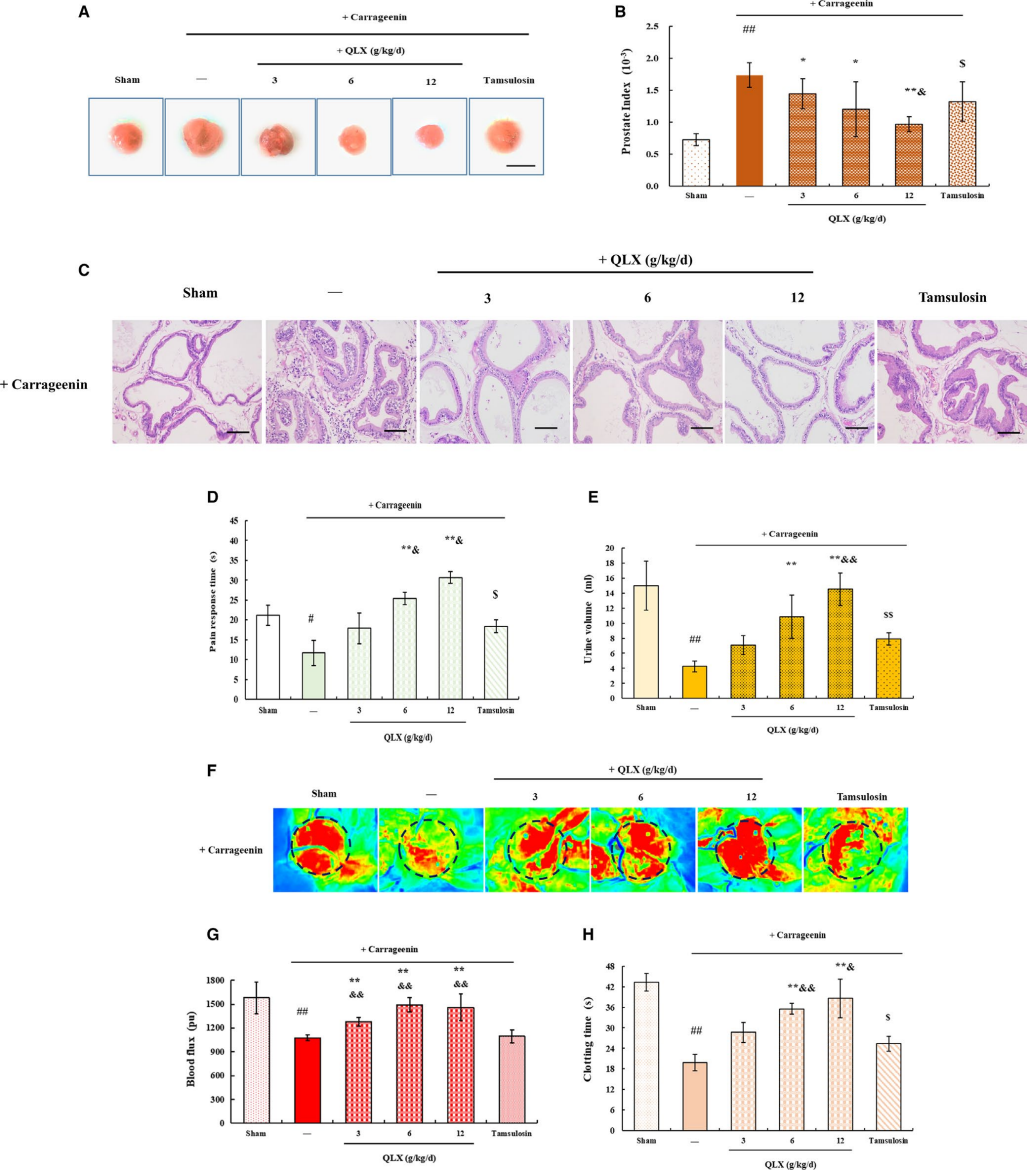
QLX reversed CGN‐induced LUTS. A, Representative photographs indicating the prostate and B, the prostate index were determined by the weight of prostate relative to the rat bodyweight. C, Representative HE staining of prostate tissue. D, The pain response time was measured on a hot plate. E, Urine was collected from rats kept for 24 h in metabolic cages. F and G, Prostate blood flow was analysed with a laser speckle contrast imager. Representative photographs and blood flow are shown. H, Clotting times. CNP and BPH were induced by direct injection of the prostate gland with CGN. QLX was administered at the indicated doses for 48 days. Control rats were injected with normal saline. Data are means ± SD, n = 3. ^#^ *P* < .05, ^##^ *P* < .01 (control vs CGN‐treated rats); *P < .05, **P < .01 (QLX‐treated vs. CGN‐treated rats); ^$$^ *P* <.01 (tamsulosin‐treated vs. oestradiol‐ or testosterone‐treated rats); ^&^ *P* < .05 (tamsulosin‐treated vs. QLX‐treated rats)

**FIGURE 6:**
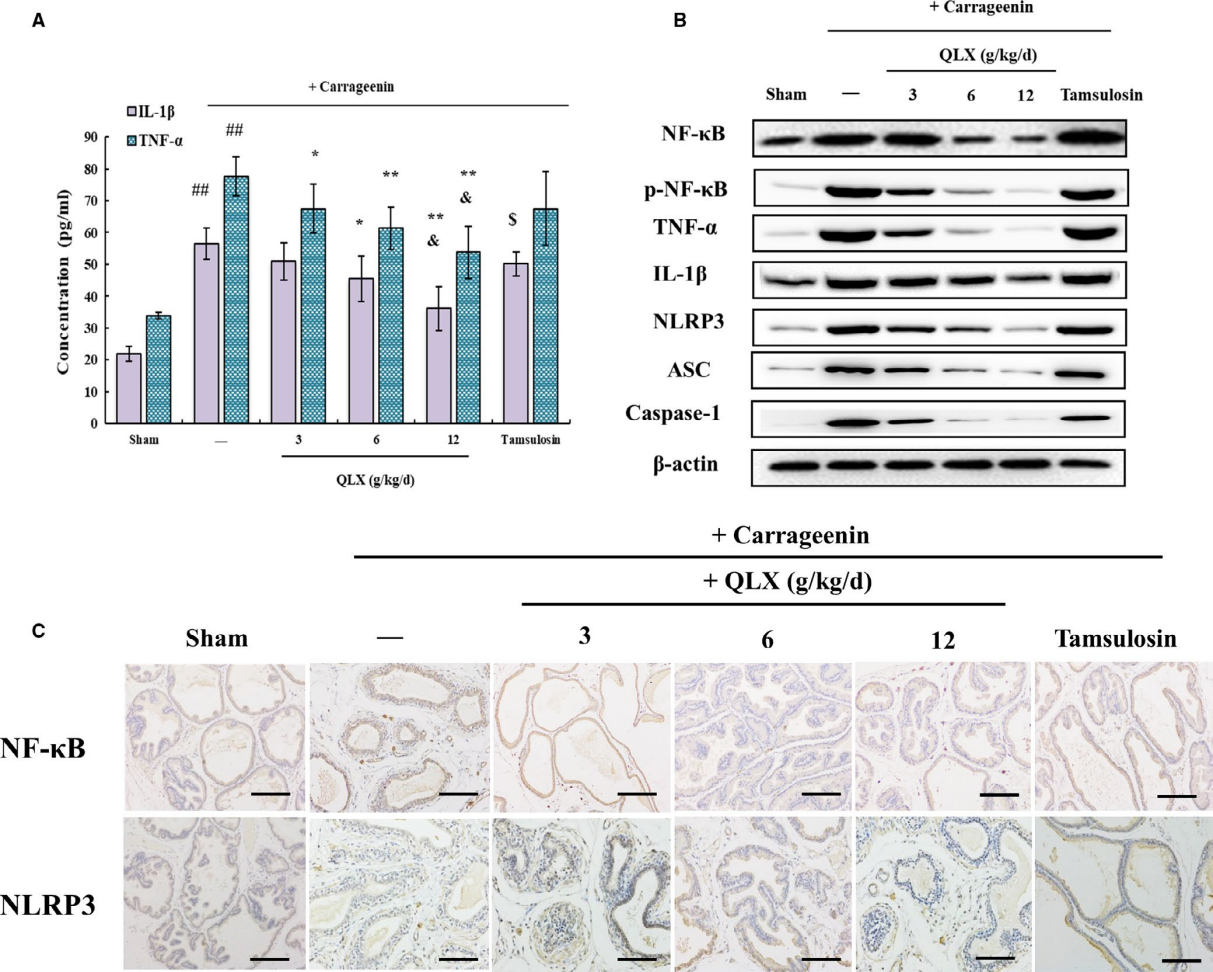
QLX exerts anti‐inflammatory effects in CGN‐induced CNP. A, IL‐1β and TNF‐β were quantified by ELISA in prostate tissue lysates. B, NF‐κB and NLRP3 expressions were assayed by IHC, and C, the expression of other proteins was assayed in Western blots. The rats were treated as in Figure 5

CGN is a polysaccharide with inflammatory activity sufficient to induce CNP and BPH in rats.^23^ In this study, CGN‐induced CNP and BPH in a rat model (Figure 1A), as shown by an increased prostate index and increased epithelial thickness (Figure 5A‐C) that were significantly reduced by QLX in a dose‐dependent manner. LUTS, including decreased pain response time (Figure 5D), decreased urine volume (Figure 5E), reduced prostatic blood flow (Figure 5F and G) and decreased clotting time (Figure 5H) in the CGN model compared with control rats, were significantly relieved by QLX. The expression of IL‐1β and TNF‐α, NF‐ κB, NLRP3, caspase‐1 and ASC was increased in prostate tissue from CGN rat models and was reduced by QLX treatment (Figure 6A‐C). Thus, QLX exerts anti‐inflammatory effects in CNP and BPH.

**FIGURE 7:**
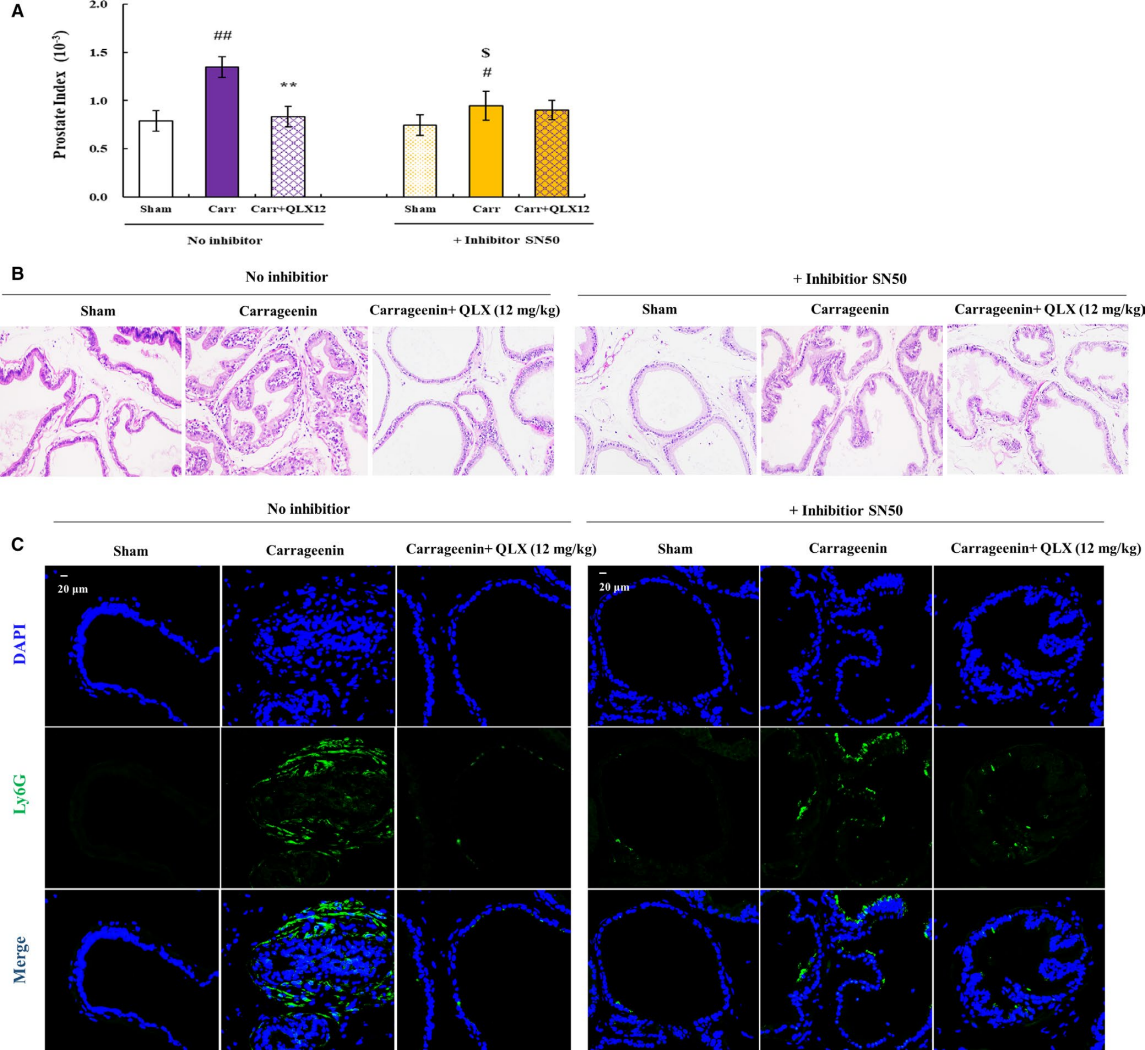
QLX exerts anti‐inflammatory effects in CGN‐induced CNP via NF‐κB. A, The prostate index was determined by the weight of prostate relative to the rat bodyweight. B, Representative HE staining of prostate tissue is shown. C, Representative IF staining photographs of Ly6G in prostate tissue are shown. SN50 was injected as described in the methods description, and CGN and QLX addition was done as described in Figure 5. ^#^ *P* < .05, ^##^ *P* < .01 (control vs CGN‐treated rats); **P < .01 (QLX‐treated vs CGN‐treated rats); ^$^ *P* < .05 (SN50‐treated in CGN rat models vs only CGN‐treated rats)

To confirm the role of NF‐ κB in the prostatic protective effect of QLX, SN50, a cell‐permeable inhibitor of NF‐κB,^24^ was injected to CGN rat models with or without QLX. CGN‐induced increased prostate index was not observed in SN50‐treated rats, and the prostate size did not further reduce after QLX treatment in CGN‐ and SN50‐treated group (Figure 7A). This suggested that the prostatic protective effect of QLX is mediated by NF‐κB, as the additional function of SN50 was not observed in QLX‐treated CNP and BPH rat model. Further, HE staining indicated that the infiltration of inflammatory cell was reduced in SN50‐treated CNP rats, and QLX did not additionally reduce the inflammatory cells in prostate tissues (Figure 7B). Ly6G, a surface marker of macrophages and monocytes,^25^ was used to quantify the infiltration of inflammatory macrophages in prostate tissues by IF, as Ly6G^+^ cells are one of the major inflammatory cells that promote prostatitis and prostate cancer.^26^ Increase in the Ly6G^+^ cells was found in CGN‐treated prostate, and QLX addition could reduce the Ly6G^+^ cells. In the case of SN50 pre‐treatment, CGN‐induced increased Ly6G^+^ cells were reduced, and QLX administration did not further reduce the Ly6G^+^ cells (Figure 7C). These suggested that the anti‐inflammatory effects of QLX in CNP and BPH were mediated by repression of NF‐ κB activation.

### 3.5 QLX has antioxidant activity in CNP and BPH

**FIGURE 8:**
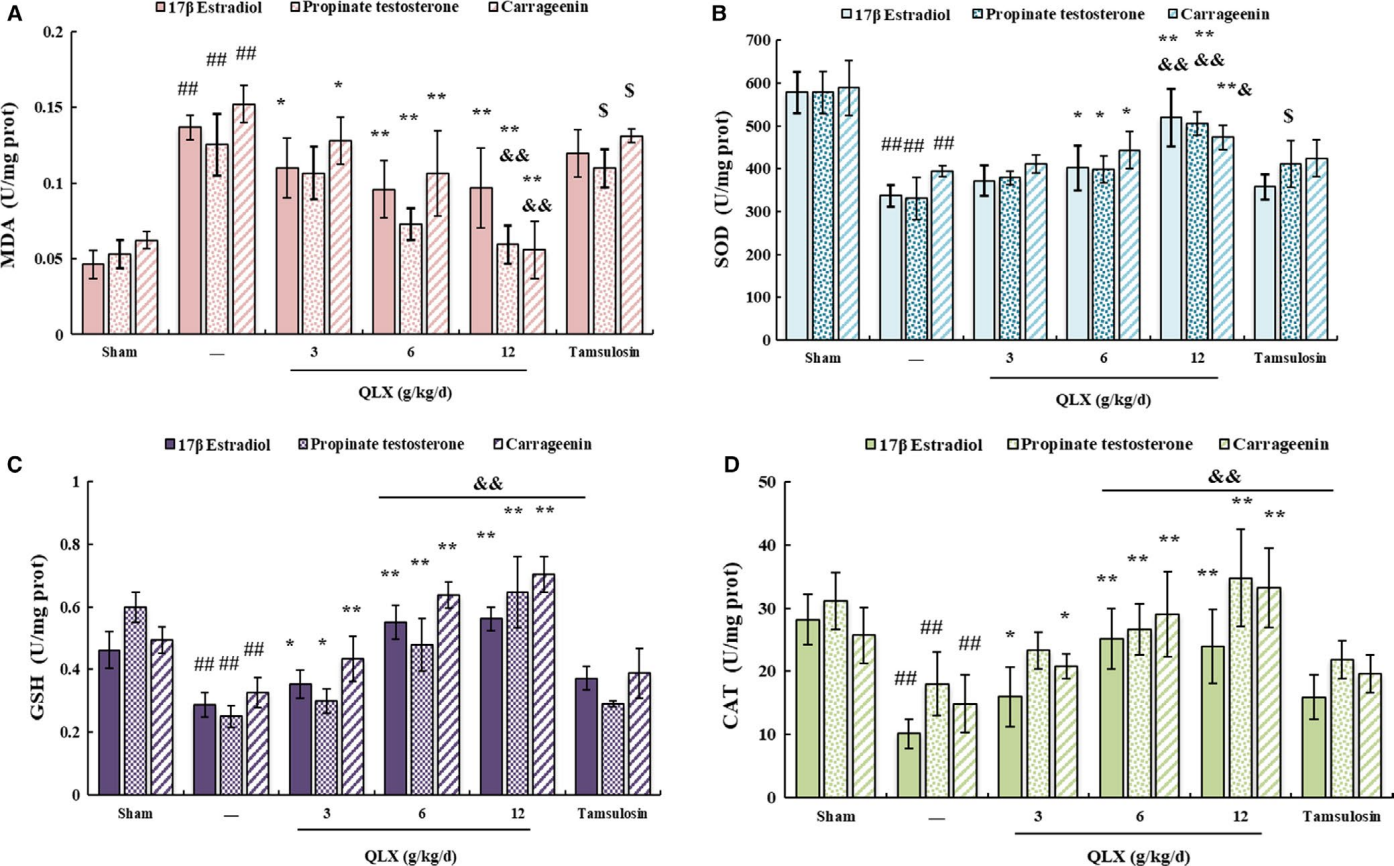
QLX exerts antioxidant effects in CNP and BPH. MDA A, SOD B, GSH C, and CAT D, were measured by ELISA. Rats were treated as described in Figures 2 and 5. Data are means ± SD, n = 3. ^#^ *P* < .05, ^##^ *P* < .01 (control vs. CGN, oestradiol‐ or testosterone‐treated rats); ^*^ *P* < .05, ^**^ *P* <.01 (QLX‐treated vs CGN, oestradiol‐ or testosterone‐treated rats); ^&^ *P* < .05, ^&&^ *P* < .01 (tamsulosin‐treated vs. QLX‐treated rats)

The polyphenol components of QLX would be expected to have antioxidant effects. Malondialdehyde (MDA) is a stable end product of lipid peroxidation that can be used as a marker of cellular oxidative stress.^27^ ELISA confirmed that MDA was increased in prostate tissue from both rat models and that QLX inhibited the increase (Figure 8A). Superoxide dismutase (SOD), catalase (CAT) and glutathione peroxidase (GSH) have antioxidant activity.^28^ The activities of all three of those enzymes were decreased in prostate tissue from both rat models compared with control rats, and the decreases were inhibited by QLX (Figure 8B‐D). The results show that QLX had antioxidant activity in the CNP and BPH models.

### 3.6 QLX enhances autophagy in CNP and BPH

**FIGURE 9:**
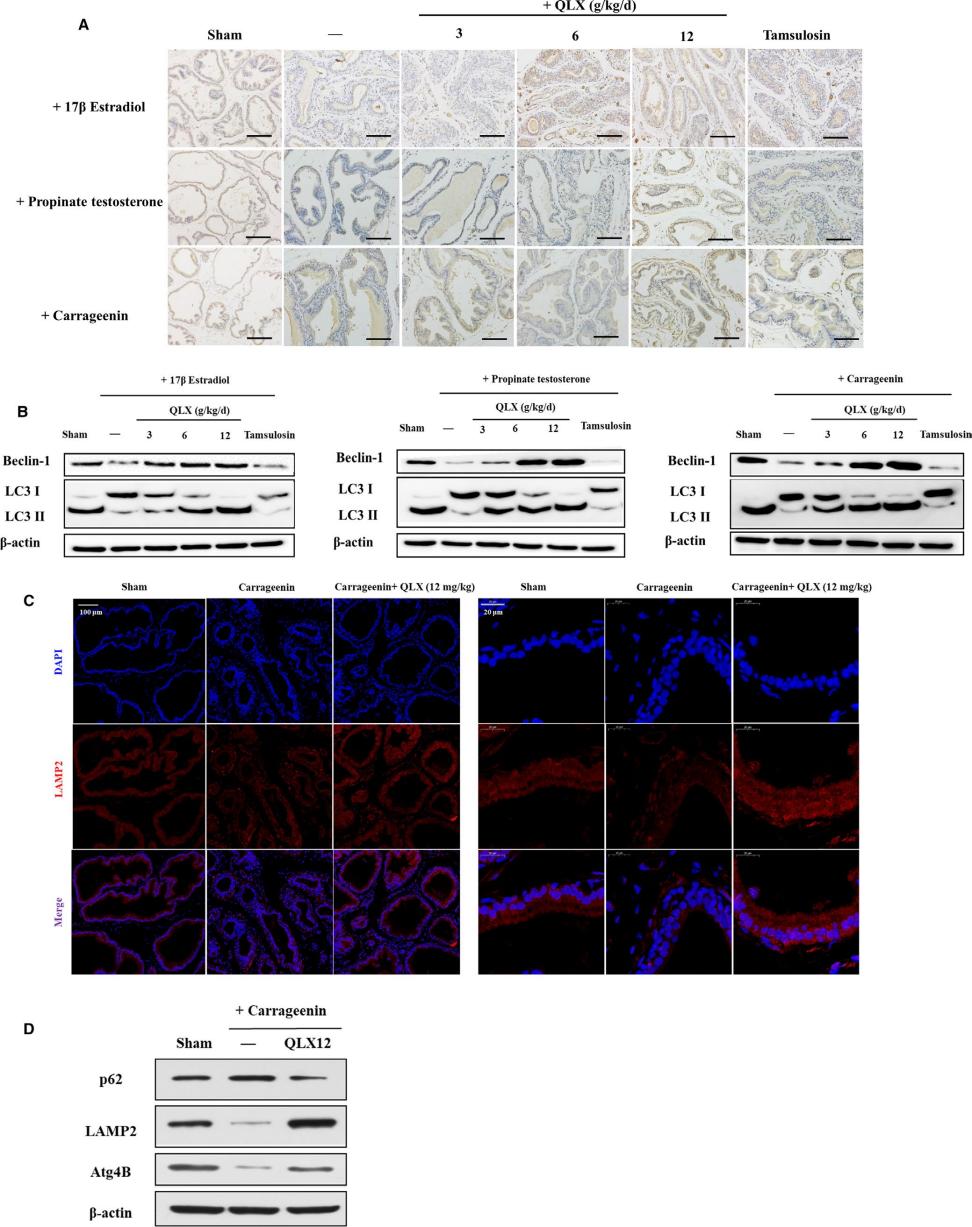
QLX repressed autophagy in CNP and BPH. A, Beclin‐1 expression was assayed by IHC. B, Beclin‐1 and LC3 I/II were assayed in Western blot in indicated protease tissues. Representative photographs are shown. C, Representative IF staining photographs of LAMP2 in prostate tissue are shown. D, The expression of p62, LAMP2 and Atg4B was measured by Western blot in indicated protease tissues. Representative photographs are shown. The rats were treated as in Figures 2 and 5

Autophagy is a key regulator of inflammasome activation,^29^ Beclin‐1 was used as marker of autophagy in this study and was quantified by IHC and Western blotting. Beclin‐1 expression in prostate tissue was decreased in both hormone imbalance–induced and CGN‐treated rats, and QLX derepressed the decreased Beclin‐1 expression (Figure 9A and B). Since autophagy is a dynamic process including formation of autophagosomes, the fusion of autophagosomes with lysosomes and lysosomes degradation.^30^ The dynamic turnover of LC3‐I to LC3‐II representing autophagic flux was measured by Western blot. LC3‐II level was down‐regulated in CGN rat models, and the extent was reversed in rats treated with QLX (Figure 9B). Similar effects of QLX on the LC3‐II/LC3‐I ratio were observed in hormone imbalance–induced CNP and BPH (Figure 9B). Atg4B could specifically cleave the C terminus of LC3 to form the LC3‐I; therefore, Atg4B plays a key role in the turnover of LC3‐I to LC3‐II during the dynamic process of autophagy.^31^ QLX also reversed CGN‐induced decreased Atg4B in prostate (Figure 9D). Moreover, the lysosomal membrane protein LAMP2 was reported to potentiate autophagic flux in brain^32^ and heart.^33^ LAMP2 expression levels were therefore quantified by IF and Western blot. Consistently, CGN‐induced decreased LAMP2 was blocked by QLX addition in prostate (Figure 9C and D). Finally, the expression of ubiquitin‐binding scaffold protein p62 was measured, as it can monitor autophagic degradation, and the expression of p62 is negatively associated with autophagic flux.^34^ CGN‐induced increased p62 was observed, and QLX addition decreased p62 expression in prostate (Figure 9 D). Thus, the results indicated that QLX can induce up‐regulation of autophagy in both CNP and BPH rat models.

## 4 DISCUSSION

In this study, QLX significantly improved LUTS in hormone imbalance– and CGN‐induced CNP and BPH rat models. The constituents of QLX include numerous polyphenols and polyphenolic metabolites such as caffeic acid, gallic acid, *p*‐coumaric acid, protocatechuic acid, neochlorogenic acid and chlorogenic acid. Dietary polyphenols are known to benefit management of BPH and CNP.^35^, ^36^, ^37^ The evidence suggested that QLX exerts a prostatic protective function and might be used to relieve LUTS associated with CNP and BPH.

QLX significantly inhibited the expression of pro‐inflammatory cytokines, which is controlled by checkpoints including transcription, maturation and release.^38^ Activation of NF‐κB in prostate cells of BPH patients was reported to be associated with increased disease severity.^39^ Gallic acid, a major chemical component of QLX, was reported to inhibit NF‐κB expression and activation followed by down‐regulation of IL‐1, ILI‐6, IL‐12, IL‐17 and IL‐23, TGF‐β and TNF‐α expression in ulcerative colitis.^40^ Inhibition of the up‐regulation of pro‐inflammatory cytokine expression by QLX might also be dependent on NF‐κB in CNP and BPH. The maturation of IL‐1β is mediated by caspase‐1.^30^ Caspase‐1 expression was up‐regulated in the CNP and BPH models in this study, and the increase in expression was significantly inhibited by QLX. The involvement of NF‐κB indicates that QLX may have mediated the transcription and release of pro‐inflammatory cytokines in the CNP and BPH models.

NLRP3 expression in CNP and BPH models was repressed by QLX. NLRP3 is an inflammasome sensor molecule that forms a multimeric complex with caspase‐1 and adaptor proteins that controls immune responses and maintains tissue homeostasis.^41^ Inflammasome activation is preceded by priming, the observed decrease in NLRP3 expression suggests that priming step was repressed by QLX. Normally, hypoxia‐induced increase in NLRP3 expression in human prostate epithelial cells is dependent on NF‐κB.^42^ QLX might inhibit NLRP3 inflammasome priming via NF‐κB. Nutritional status and oxidative stress are key regulators of inflammasome activation.^34^ In mice, autophagy deficiency has been associated with increased IL‐1β expression in response to stimulation compared with normal cells.^26^ Rapamycin‐induced autophagy has been reported to attenuate NLRP3 inflammasome activation in CNP and BPH.^43^ It is thus possible that the increased autophagy observed in QLX‐treated rats inhibited NLRP3 inflammasome activation. The generation of ROS associated with the cellular redox state also modulates NLRP3 inflammasome activation. Inhibition of mitochondrial complex I has been shown to increase ROS generation that resulted in increased NLRP3 activation and release of pro‐inflammatory cytokines.^44^ Increased oxidative stress in human BPH tissues is positively correlated with prostate weight and prostatic inflammation.^45^, ^46^ ROS have also been found to promote NLRP3 inflammasome priming.^47^ The antioxidant activity of QLX might be required for inhibition of inflammasome priming and activation.

**FIGURE 10:**
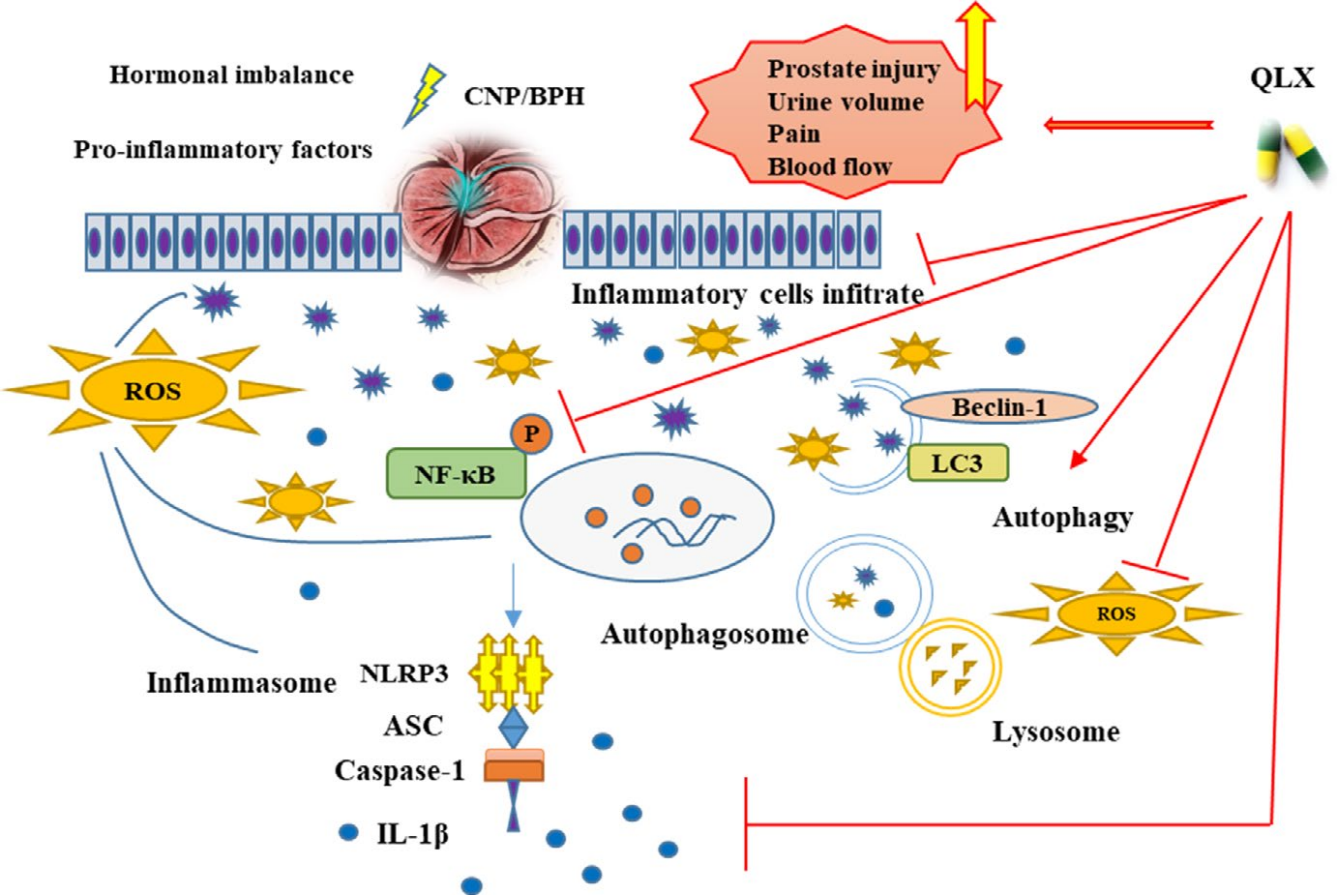
Summary of the anti‐inflammatory activity of QLX in CNP and BPH

The herbal drug QLX had anti‐inflammatory effects in hormone imbalance–induced and CGN models of CNP and BPH. QLX may have repressed the expression of pro‐inflammatory cytokines and NLRP3 inflammasome priming by inhibiting NF‐κB activation and NLRP3 inflammasome activation (Figure 10). The anti‐inflammatory effects of QLX may be helpful for the treatment of LUTS in CNP and BPH patients.

## CONFLICT OF INTEREST

Tian F., Yao Y., Meng Z., Fan S. and Zhang Y. belong to the Shandong Hongjitang Pharmaceutical Group Co., Ltd., which has the patent of Qianliexin capsule.

## AUTHOR CONTRIBUTIONS

**Linghe Zang:** Conceptualization (lead); Data curation (lead); Formal analysis (lead); Investigation (lead); Methodology (lead); Writing‐original draft (lead); Writing‐review & editing (lead). **Fangyuan Tian:** Data curation (equal). **Yuancheng Yao:** Methodology (equal); Software (equal). **Yiran Chen:** Data curation (supporting). **Yuan Shen:** Data curation (equal); Methodology (equal). **Mingyu Han:** Investigation (equal); Methodology (equal). **Zhaoqing Meng:** Formal analysis (equal). **Shengci Fan:** Funding acquisition (equal). **Xinyi Zhang:** Investigation (equal); Visualization (equal). **Tian Cai:** Data curation (equal). **Qi Gao:** Data curation (equal). **Yu‐wei Zhang:** Funding acquisition (equal). **Jincai Lu:** Funding acquisition (equal); Project administration (equal); Writing‐original draft (equal); Writing‐review & editing (equal).

## Supporting information

Supplementary Material
