# Supplementary material for: Qianliexin capsule exerts anti‐inflammatory activity in chronic non‐bacterial prostatitis and benign prostatic hyperplasia via NF‐κB and inflammasome
Source: J Cell Mol Med. 2021 May 13;25(12):5753–68. doi: 10.1111/jcmm.16599 (PMC8184730; doi:10.1111/jcmm.16599)
Supplement: Supplementary file 1 — Supplementary Material [file JCMM-25-5753-s001.docx]

**Supplement Figure**

A


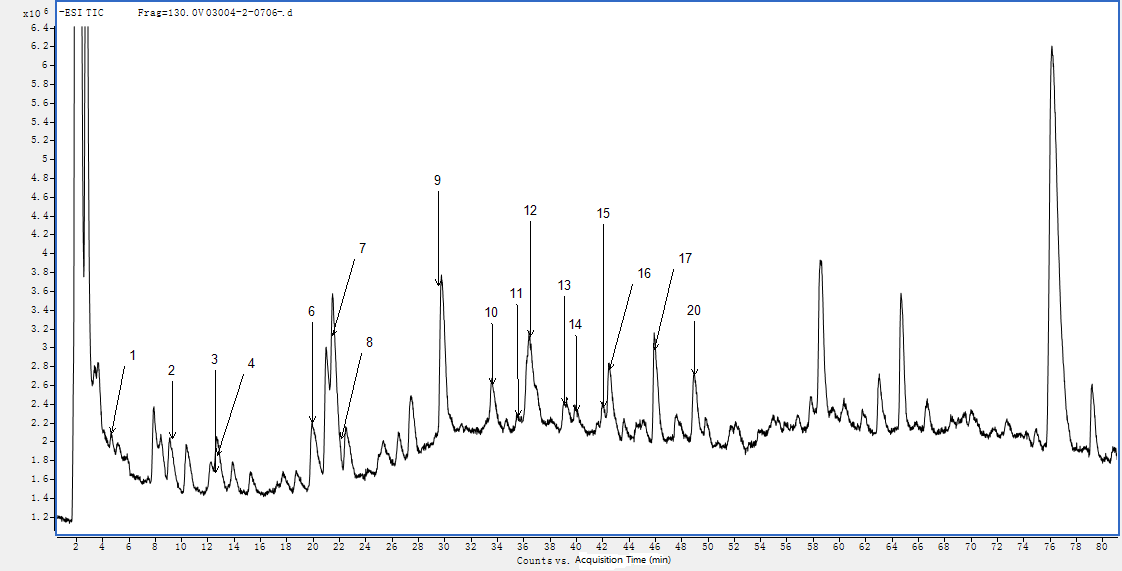


Negative ion mass spectrum

B


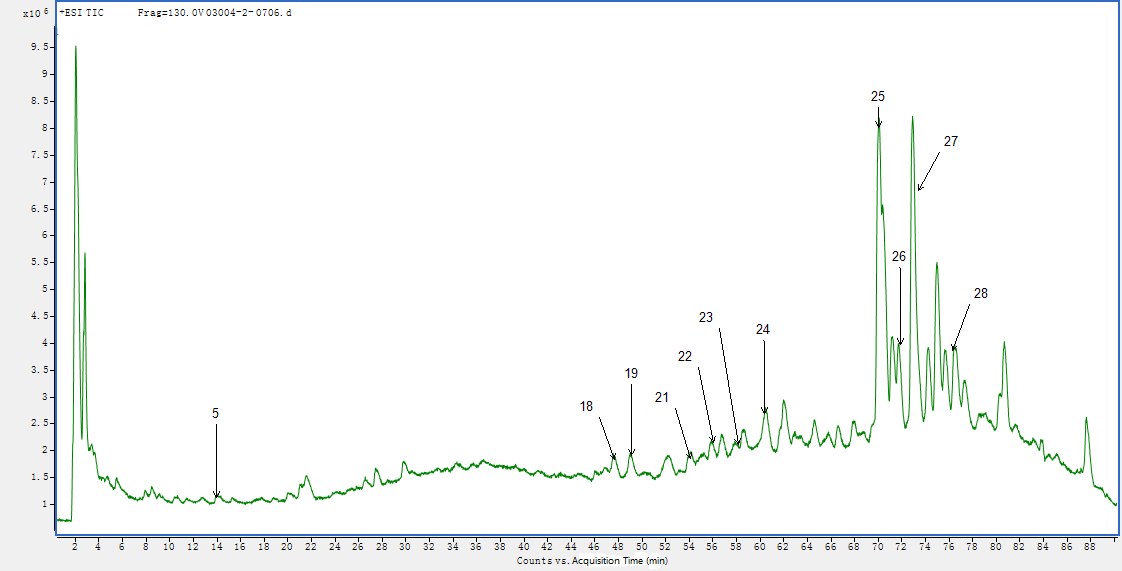


Positive ion mass spectrum

C

Supplement Figure. UHPLC fingerprint chromatograms of the QLX extracts that were obtained by negative ion mass spectrum (A) and positive ion mass spectrum (B). (C) Chemical structures of the 28 components identified in QLX.
